# A Treatise on the Physical Cause of the Death of Christ, and Its Relation to the Principles and Practice of Christianity

**Published:** 1847-10-01

**Authors:** 


### A Treatise on the Physical Cause of the Death of Christ, and its relation to the Principles and Practice of Christianity. By *William Stroud*, M.D. Pp. 496. Hamilton and Adams: London, 1847.

This is a remarkable work—remarkable alike for the subject of which it treats, and for the admirable manner in which it is written. It displays a great deal of professional research, and, at the same time, no ordinary scholarship in biblical as well as in classical literature. Everywhere it breathes a spirit of pure and elevated religious feeling; the main object and desire of the writer being evidently the simple discovery of Truth, and its practical application to the welfare of himself and others. He has sought to do good rather than to acquire fame, to improve and edify the heart of his readers even more than to enlighten and inform their understandings. His volume is obviously the fruit of long and patient study, and of deep and solemn meditation, such as the importance of its theme demanded. Dr. Stroud has read much, but he has thought more. While he has, with most praiseworthy diligence, perused the writings of very numerous authors on most of the topics connected with his argument, he has always kept his mind unfettered by any mere human authority; and thus he never fails to exercise an independent judgment of his own. His chief study has been the Holy Scriptures themselves; and to them, and to them alone, he appeals as the source of all divine instruction. So much for the general tone and and character of the present work. And here it may be necessary to guard the reader from supposing that it is more curious than instructive, more likely to attract by the singularity of its views than convince by the soundness of its reasonings, or edify by the piety of its reflections. The title of the book may possibly lead some readers to imagine that it deals in speculations that are, to say the least of them, profitless. But this is not the case. It has been no mere curiosity of learning that prompted the author to engage in the enquiry, no idle exercise of his mind, no love of notoriety or distinction. Dr. Stroud has had a much higher and more worthy motive to stimulate him at first, and to direct him throughout his labours. And, has he not found his reward? We feel confident that he has. His labour has not been in vain. He has anxiously sought for the truth; and a striking development of it has been manifested to him. The result of his researches has been, if not the discovery, at least the happy elucidation of certain points in the sacred narrative of our Lord's sufferings which were, before, imperfectly understood, and the right appreciation of which serves to throw very considerable light upon various parts of Holy Writ. This was the chief recompense he sought for; and, trifling though it may seem in the eyes of the multitude, it has a power of inward enjoyment and self-satisfaction which they are little aware of.

The chief object of the work is stated by Dr. Stroud himself to be—

"To demonstrate an important physical fact connected with the death of Christ, and to point out its relation to the principles and practice of Christianity; but, although the subjects discussed and the conclusions deduced from them are, it is hoped, of no inconsiderable value in a devotional point of view, the treatise itself is rather argumentative than sentimental, and more concerned with the foundation of evangelical religion than with its superstructure. The fact is not indeed now announced for the first time, having been more or less correctly anticipated by several pious and excellent writers during the last century; but, as in matters of such solemn import conjecture and probability are not a sufficient ground for conviction, the author has laboured to supply a demonstration of the fact, which he trusts will be found both new and satisfactory. He has accordingly been careful not to assume anything which is not generally acknowledged; and has supported every point of the argument with proofs and evidences so combined, as apparently to leave no other alternative than that which is here maintained. Should the attempt have been successful, it will furnish a fresh proof of the value of inductive reasoning; which, like a sounding-line let down into the ocean of time, has thus, from the depth of eighteen hundred years, brought up to the surface a pearl of great price."—*Preface*, p. vi.

As the argument and its illustrations are almost entirely of a Physiological nature, and as medical men must therefore be better able to estimate their value and their force than others, we can have no hesitation in inviting the attention of our readers to the consideration of a volume that, in our judgment, is so creditable to its author, not less as an enlightened physician than as a sincere Christian. The reasoning indeed is mainly physiological, but its application is essentially spiritual; the data on which it rests are drawn from medical science, but the great lesson which the whole is designed to teach is to impress the mind with a deeper and a more devout sense of that divine love that moved the Saviour to endure the mysterious agonies of the garden and of the cross, in working out the Redemption of the human race. As a matter of course, it is the medical argument that will almost exclusively engage our attention. And here we would remark that, whatever opinion may be formed of the reasonings advanced in the volume before us, and whether its conclusions be adopted or not, no one, we feel assured, will rise from its perusal without having the utmost esteem for its pious and talented author. In his hands, Medicine becomes the handmaid of Religion; the discoveries of physical Science are consecrated by being laid on the altar of heavenly Truth. And is not the example worthy of imitation? Are medical men always to shrink from even so much as making any allusion to the blessed pages of the Bible? Is every branch of learning to be studiously examined and discussed, and may not a word be ever said of one great end and object of all instruction; viz. the elevation of the heart, the spiritualising of the affection? Might it not be well if our thoughts and feelings were, at times, withdrawn from the incessant attention that is paid in the present age to the merely material portion of our professional pursuits, and occasionally directed to the contemplation of subjects which interest us, in common with our fellow-creatures, as sojourners in a world where every thing proclaims that we are but as links of a mighty chain whose ends are hid in impenetrable darkness, and that the great object of our lives is for purposes, the fulfilment of which reaches on to another state of existence?

No set of men, it must be obvious, have so many advantages, derivable from their education and from the nature of their engagements, in following out the study of many questions appertaining to the moral happiness and welfare of mankind, as the members of the medical profession. To them, more frequently than to others, it is given to observe the mutual relationship between mind and matter, between spirit and body; and to witness the effects of this relationship either for good or for evil, for happiness or for misery to the individual. They have peculiar facilities of watching, if not of explaining, the reciprocal actings and re-actings of the corporeal and psychical portions of our compound nature; for their aid is as often invoked to "minister to a mind diseased," as to relieve the sufferings of the body, and they are frequently called upon to soothe "the troubles of the brain," though they cannot "cleanse the foul bosom of that perilous stuff which weighs upon the heart."

May the following remarks be made profitable to some readers by awakening their attention, more than perhaps it has yet been, to a class of subjects that well deserve to be thought upon and pondered by all! The first part of our observations is intended to illustrate the effects of certain strong passions on the bodily frame; in the second, we shall point out the application which Dr. Stroud has made of this enquiry, to explain what he terms "the physical cause" of the death of the Redeemer.

The influence of intense mental emotions, whether of an exciting or of a depressing nature, on the actions of the Heart and blood-vessels and on the vital fluid that permeates through them, has been recognised by medical writers in all ages. A few authorities will suffice:

"It is observed by Baron Haller, the father of modern physiology, that excessive grief occasions palpitation, and sometimes sudden death; that the corporeal effects of anger and terror are nearly alike, including increased strength, and violent motions both in the heart and throughout the body, and producing *bloody sweats*, and other kinds of hæmorrhage.*—'Anger'—says Senac—'has in certain cases torn torn the fibres of the heart, and even opened the ventricles. It is not therefore extraordinary that it should be followed by palpitation, and accordingly, various physicians have observed such a result...... But fear and terror are not less powerful causes, especially when they seize suddenly. In that case the nerves act with violence on the heart, and derange the order of its movements. The blood is at the same time propelled in these passions by a general shock, or commotion of all the parts of the body: it therefore necessarily accumulates in the two trunks of the venæ cavæ, rushes into the auricles, and overcharges them, as well as the ventricles. Here then are two causes, one the consequence of the other, which, as is proved by numerous examples, produce palpitation. Dilatations are, as we have already stated, frequent results of fits of passion. Grief and sadness do not act so suddenly, nor with equal force; but, as we have said, these secret and silent passions induce similar disorder.'†—'If any one'—remarks Corvisart—'can seriously deny, or even doubt the fatal physical influence of the passions on the heart, let it suffice him to know that a fit of anger may produce rupture of the heart, and cause sudden death......... Complete rupture of the heart has rarely been observed in the sound state of this organ: some examples may however be cited of this lesion, in consequence of a violent effort, a fit of anger, an epileptic paroxysm, &c......... But of all the causes capable of producing organic diseases in general, and more especially those of the heart, the most powerful beyond dispute are mental affections....... No mental affection can indeed be experienced without the movement of the

* "Haller, Element. Physiog. Corp. Human., vol. v., pp. 50, 583, 586, 587."

† "Senac, Traite du Cœur, vol. ii., p. 515."

heart being either augmented, accelerated, retarded, weakened, or disturbed, without its force in fact being increased, enfeebled, or almost annihilated. Pleasure, pain, fear, anger, in short all the powerful passions, cause the heart to palpitate, to beat more or less frequently, strongly, slowly, or regularly, or to suspend its action momentarily, sometimes even mortally.*" P. 78.

The effects of Terror on the organs of circulation, &c. are thus described by Dr. Alex. Chrichton.†—" The heart is thrown into greater and more violent action than usual, but the arterial system, so far from corresponding with it in a general sense, is either rendered torpid at its extremities, or else is affected with a spasm; a sudden paleness spreads itself over the countenance, the lips lose the coral tint, and the whole body of the man seems to shrink into a smaller compass, a tremor agitates his whole frame, and he feels as if he had suffered a great diminution of strength......... It happens now and then, when the whole play of the mental faculties is as it were destroyed by the impression of the dreadful object, and no possibility of escape appears, that, volition being then without a stimulus, a person drops down on the earth, as if suddenly bereft of all his animal powers."

The same writer has well depicted the effects of Grief and Sorrow:

" The general corporeal effect of all the modifications of grief and sorrow is a torpor in every irritable part, especially in the circulating and absorbent system: hence the paleness of the countenance, the coldness of the extremities, the contraction and shrinking of the skin and general surface of the body, the smallness and slowness of the pulse, the want of appetite, the deficiency of muscular force, and the sense of general languor which overspreads the whole frame. As the action of the extreme branches of the arterial system is greatly diminished, the heart, and aorta and its larger vessels, and the whole system of the pulmonary artery become loaded and distended with blood. The painful sense of fulness which this occasions gives rise to a common expression, which is in some degree descriptive of what really exists. In sorrow the heart is said to be *full*, and in deep sorrow it is often said to be *like to burst*. A sense of oppression and anxiety, a laborious and slow respiration, and the remarkable phenomena of sobbing and sighing, naturally arise from this state of torpor and retarded circulation." P. 82.

It is rather the suddenness and violence, than the nature or kind, of the mental emotion that is apt to produce the consequences now described. Death has been known to be produced by excess of unexpected joy, as well as by overpowering grief or terror. We read that Sophocles died from elation, at his triumph in a contest of honour. Aulus Gellius tells us of a man, who suddenly expired on hearing of his three sons being crowned as victors on the same day; and Livy mentions the case of an aged matron who, having been in the depth of distress from the tidings of her son's being slain in battle, died in his arms from the excess of joy at his safe return. In these and such-like cases, the death is, as our author observes, probably owing to some sudden lesion, functional or organic, of the heart. Zimmerman says that Philip V. died suddenly on being told that the Spaniards had been defeated; on opening his body the heart was found ruptured.‡

* " Corvisart, Sur les Maladies du Cœur, &c.; Discours Preliminaire, p. xli. pp. 259, 369, 370."

† On Mental Derangement. Vol. II., pp. 119—121, &c.

‡ On Experience in Physic. Vol. II., p. 268.

But, before we examine this subject more minutely, let us turn our attention to that rare certainly, but nevertheless perfectly well-authenticated, effect of strong mental emotion upon the secretion of the skin; we allude to the remarkable phenomenon of *bloody sweat.* The following instances are adduced by Dr. Stroud, in the way of illustration.

"The eminent French historian De Thou mentions the case of—'an Italian officer who commanded at Monte-Maro, a fortress of Piedmont, during the warfare in 1552, between Henry II. of France and the emperor Charles V. This officer, having been treacherously seized by order of the hostile general, and threatened with public execution unless he surrendered the place, was so agitated at the prospect of an ignominious death, that he sweated blood from every part of his body.'—The same writer relates a similar occurrence in the person of a young Florentine at Rome, unjustly put to death by order of Pope Sixtus V. in the beginning of his reign, and concludes the narrative as follows. 'When the youth was led forth to execution, he excited the commiseration of many, and through excess of grief, was observed to shed bloody tears, and to discharge blood instead of sweat from his whole body; a circumstance which many regarded as a certain proof that nature condemned the severity of a sentence so cruelly hastened, and invoked vengeance against the magistrate himself, as therein guilty of murder.' Amongst several other examples given in the Ephemerides, of bloody tears and bloody sweat occasioned by extreme fear, more especially the fear of death, may be mentioned that of—'a young boy who, having taken part in a crime for which two of his elder brothers were hanged, was exposed to public view under the gallows on which they were executed, and was thereupon observed to sweat blood from his whole body.'—In his Commentaries on the four Gospels, Maldonato refers to—'a robust and healthy man at Paris who, on hearing sentence of death passed on him, was covered with a bloody sweat.'—Zacchias mentions a young man who was similarly affected on being condemned to the flames. Schenck cites from a martyrology the case of—'a nun who fell into the hands of soldiers; and, on seeing herself encompassed with swords and daggers threatening instant death, was so terrified and agitated, that she discharged blood from every part of her body, and died of hemorrhage in the sight of her assailants;'*—and Tissot reports from a respectable journal that of 'a sailor who was so alarmed by a storm, that through fear he fell down, and his face sweated blood, which during the whole continuance of the storm returned like ordinary sweat, as fast as it was wiped away.' "† P. 88.

Haller, in more than one passage of his physiological writings, has alluded to the exsudation of a bloody fluid from the cutaneous vessels. "Mental emotions," says he in one place, "have been known to produce extraordinary changes in the secretions, so that blood and bile have been forced out from the vessels of the skin."‡ Perhaps one of the most striking instances on record of the oozing of a sanguineous fluid from the surface

---

* "Ephemerid. Acad. Natur. Curios. Ann. 2, p. 34;—Dec. ii. Ann. 10, p. 354;—Dec. iii. Ann. 1, Append. pp. 124, 125;—Ann. 7 and 8, Append. p. 124;—Ibid. edit. 2da, vol. i. p. 84;—vol. viii. p. 184:—Thuanus, Hist. sui Temp. vol. i. p. 373; vol. iv. p. 300; Joannes Maldonatus, Comment. in quatuor Evangelist. p. 601;—Paulus Zacchias, Quæstiones Medico-legales, lib. iii. p. 154;—Joannes Schenck à Grafenberg, Observ. Medic. &c. lib. iii. p. 458."

† Tissot, Traité des Nerfs, &c. pp. 279, 280.

‡ Elementa Physiologiæ Corp. Hum. vol. v., p. 50.—Primæ Lineæ Physiologiæ, p. 126.

occurred in the person of Charles IX. of France, who died in the 25th year of his age. We read in De Mezeray, the historian, that, "after the vigour of his youth and the energy of his courage had long struggled against his disease, he was at length reduced by it to his bed at the castle of Vincennes, about the 8th of May (1574). During the last two weeks of his life, his constitution made strange efforts. He was affected with spasms and convulsions of extreme violence. He tossed and agitated himself continually, and his blood gushed from all the outlets of his body, *even from the pores of his skin;* so that, on one occasion, he was found bathed in a bloody sweat." Voltaire, in alluding to this narrative, adds:—"This malady, of which there are some examples, is the result either of excessive fear, furious rage, or of a violent and melancholic temperament."* Besides this, and other cases already mentioned, Dr. Stroud quotes the following one, related in the French "Transactions Medicales" from a former number of this Journal. It stands thus:—

"A young woman aged twenty-one years, irregular in menstruation, and of indolent habits, and obstinate temper, had been much irritated by some reflections made by her parents, on account of her abjuring the Protestant religion. She left her paternal roof, and after wandering about for some time, took up her residence in an hospital. She was then suffering violent attacks of hysteria, attended with general convulsions, and exquisite sensibility in the pubic and hypogastric regions. After paroxysms of hysteria, which sometimes lasted twenty-four or thirty-six hours, this female fell into a kind of ecstacy, in which she lay with her eyes fixed, sensibility and motion suspended. Sometimes she muttered a prayer, but the most remarkable phenomenon was an exudation of blood from the cheeks and the epigastrium in the form of perspiration. The blood exuded in drops, and tinged the linen. The cutaneous surface appeared injected in those parts whence the blood escaped, being red, and showing a network of arborescent vessels. This bloody perspiration took place whenever the hysteric paroxysm lasted a considerable time. This state continued for three months, and ultimately gave way, it is said, to local bleeding, . . . . together with strong revulsive measures.† P. 382.

This case will probably call to the recollection of many of our readers the strange stories about certain "estaticas," who were, a few years ago, (and perhaps still are) exhibited in some nunnery in Italy to the edification of the credulous. We gave a short account of the phenomena alleged to be witnessed in these deluded young women, in our number for October 1845. It is worthy of remark that they were all subject to violent convulsive attacks, and were evidently labouring, at the same time, under the influence of intense mental excitement. Their malady was doubtless one of the most distressing forms of Hysterical disease, just as in the instance of the girl whose case has been related above.

We now proceed to shew, as already intimated, that intense mental emotion, more especially when it is of an exciting nature, may suddenly induce a mortal lesion of the Heart, and that this lesion has been, in some cases at least, an actual rupture of this vital organ. The following extracts

---

* De Mezeray, Histoire de France, vol. iii.—Voltaire, Œuvres completes, vol. xviii.

† Medico-Chirurgical Review for Oct. 1831, p. 496.

from the writings of two distinguished writers of our own day, may be appropriately quoted upon this point.

" 'It is generally in the left ventricle," remarks Dr. Hope, "that the rupture [of the heart] takes place; a circumstance which at first appears remarkable, since this ventricle is the stronger, but for the same reason it contracts more energetically, and . . . it is only strong muscles which undergo rupture from the energy of their own contraction. Hence rupture of the auricles is much more rare than that of the ventricles. The exciting causes of rupture are generally considerable efforts, paroxysms of passion, external violence, as falls, &c. . . . . . . Rupture of the heart or great vessels into the pericardium is not always immediately fatal, as a solid coagulum or a fibrinous concretion has in several instances been known to arrest the hemorrhage for a few hours. Of ten cases mentioned by Bayle, eight died instantaneously, one in about two hours, and another in fourteen.'—Amongst the causes of rupture of the heart Dr. Copland enumerates,—'violent mental emotions, especially anger, fright, terror, unexpected disappointment, distressing intelligence abruptly communicated, anxiety, &c., sudden and violent muscular efforts, and laborious or prolonged physical exertions of any kind, particularly in constrained positions. . . . . . In some cases,'—he observes,—'inexpressible anxiety and pain have been felt in the præcordia and epigastrium, with cold extremities, and cramps, shortly before dissolution. In the majority rupture has produced instant death, but in some this has not been the case. . . . . . In most of the cases in which the rupture is preceded by violent pain, M. Ollivier thinks that it is produced gradually from the successive laceration of several layers or fasciculi of muscular fibres, and that the pericardium becomes only gradually distended by the effused blood. Where the laceration and aperture are at once large, a copious effusion instantly occurs, fills the pericardium, and abolishes the contractions of the organ."* P. 90.

It must be confessed, indeed, that there are not many instances on record in which sudden death from mental emotion has been *proved*—by dissection, we mean—to have been owing to Rupture of the Heart. In many such cases, no *post-mortem* examination has been made. Nevertheless, it may be reasonably conjectured that this mortal lesion is not so extremely rare as has been generally supposed. Dr. Stroud thinks it highly probable that the death of Palmer, about the close of the last century, while enacting the part of the Stranger in the play of that name on the boards of the Liverpool theatre, was from this cause. The circumstances were these. For some time previously, he had been labouring under the most profound grief, in consequence of the death of his wife and a favourite son, and he had struggled to bear up against it, although he felt that his mental sufferings would very shortly bring him to the grave. "On the morning of the day, he was much dejected; but exerted himself with great effect in the first and second acts of the play. In the third act, he shewed evident marks of depression; and in the fourth, when about to reply to the question of Baron Steinfort relative to his children, he appeared unusually agitated. He endeavoured to proceed, but his feelings overcame him. The hand of death arrested his progress, and he fell on his back, heaved a convulsive sigh, and instantly expired without a groan. . . . . Dr. Mitchell and Dr. Corry gave it as their opinion,

* " Dr. Hope, On the Diseases of the Heart and great Vessels, pp. 198, 199;—Dr. Copland, Dict. of Practical Medicine, Part v., p. 224."

that he certainly died of a *broken heart*, in consequence of the family afflictions which he had recently experienced."

The expression here used, it is very reasonably imagined, may have been literally, as well as metaphorically, true. We shall presently adduce several examples in corroboration of this idea. Meanwhile, it may not be uninteresting to remark, *en passant*, that the language of every nation has recognized, so to speak, its strict physiological truth. Hence, as well observed by our author, "although the term—*broken heart*—is not always used literally, but often in a figurative sense to denote intense or perhaps mortal sorrow, it was no doubt originally derived from the actual fact, either accidentally observed, or sagaciously conjectured by poets and moralists, habitually engaged in the study of the human passions and of their influence on the bodily frame." Shakespeare, with that intuitive penetration of genius which was so pre-eminent in him beyond all other writers, has repeatedly alluded to this cause of sudden death from agony of mind. An instance or two must suffice :

"Give sorrow words: the grief that does not speak
Whispers the o'erfraught heart, and bids it break; and by a bold poetical license, Marc Antony is made to represent the death of Julius Cæsar as occasioned, not by the daggers of his conspirators, but by his anguish of soul on seeing his friend Brutus in their number:

"Ingratitude, more strong than traitor's arms,
Quite vanquished him : then burst his mighty heart,
And, in his mantle muffling up his face,
Even at the base of Pompey's statue
Which all the while ran blood, great Cæsar fell."

For other passages of a similar bearing, the curious reader must consult Dr. Stroud's work : proceed we to more matter-of-fact details.

Although actual Rupture of the Heart is unquestionably a rare occurrence from mental emotion or from any cause whatsoever,* it is not a difficult thing to prove, from the concurrent testimony of numerous writers, the direct influence of strong passion in inducing less serious lesions of this vital organ. Harvey relates the case of a man who, under the long-continued working of smothered indignation and revenge, fell into a scorbutic and hæmorrhagic state, attended with extreme oppression and pain of the chest, owing to an immense enlargement of the heart and principal arteries, which was entirely the result (it was believed) of mental emotion. "Had this emotion," Dr. Stroud remarks, "been more intense, it is easy to conceive that, instead of a slight oozing of blood from the cutaneous vessels, and a mere enlargement of the central organs of circulation, the result would have been bloody sweat, and rupture of the heart."

Dionis has related an interesting case of an officer affected with serious disease of the heart, attributed by the patient himself to the strong efforts

---

* It has been asserted by some writers that Rupture of the Heart never takes place in a normal state of the organ; but this statement has been denied by Portal and Rostan, in whose writings are recorded numerous observations which prove the contrary, and which serve to shew that rupture of the left ventricle may take place without any coexisting alteration of its muscular tissue.

which, several years before, he had used to suppress a violent fit of anger. On dissection, the right auricle was found as big as the head of a new-born child, and contained nearly a pint and a half of blood, the greater part of which was coagulated. "In like manner, Tissot quotes from Viridet the case of a merchant, who, in consequence of violent grief, was seized with constriction and severe pain of heart, terminating in death. On inspection of the body,—'the heart was found twice as large as it should have been, and the whole of its left cavity filled with blood strongly coagulated.' —In another merchant, aged sixty-two years, who suddenly died of grief, Bonet states that the heart and lungs were found greatly distended with blood which, in the right ventricle, was almost entirely coagulated. Of the separation of the blood in some of these cases into its constituents, the same author gives two examples.—'A paralytic orphan girl, seventeen years of age, suddenly died of suffocation without any obvious cause. On dissecting the body, I found the heart of twice the usual size, its auricles very large, and like the veins and arteries, much distended with water and black clotted blood....... In a soldier who suddenly died after long-continued grief, whilst all the other viscera were healthy, the pericardium was found to contain not only water, but also much coagulated blood.' "*

In reference to this last case, Morgagni has remarked:—"Although you will see it repeated in a note that the heart was loaded both with *blood and water*, it is by no means necessary that you should believe this water to have been any other than the serum of the blood separated from the coagulated part, as not unfrequently happens to a considerable amount."

This remark of Morgagni leads us, by a natural transition, to the consideration of a topic connected with rupture of the heart, which will be found to have a most important bearing on the general argument of our present enquiry. It deserves, therefore, to be examined at some length.

The separation of the blood into its solid and fluid constituent parts seldom or never takes place completely while it remains within the heart and the vessels, unless, indeed, their structure or conformation be seriously altered, as in cases of aneurismatic enlargement, &c. It is commonly either wholly liquid or wholly solid, or a portion of liquid blood is intermixed with pale or dark-coloured coagula; but its distinct or complete separation into serum and crassamentum—or, to use popular language, into *blood* and *water*—is scarcely ever met with. The attention of anatomists has hitherto been little directed to this subject. Two gentlemen, however, distinguished alike for the extent of their enquiries and the accuracy of theit observations, have very fortunately, within the last few years, published some remarks upon it; we allude to Dr. John Davy and Mr. Paget. The results of their labours may be briefly stated to be as follow. Of 164 cases in which the state of the blood was noted by the former of these gentlemen, it was found "coagulated and containing fibrinous concretions, in 105;—coagulated and broken up, as if by the contractions of the heart,

* Harvæus, Opera, pp. 127, 128;—Dionis, Anatomy of Human Bodies, pp. 270, 445—451;—Tissot, Traité des Nerfs, &c. p. 361;—Bonetus, Sepulchretum, vol. i. pp. 585, 887, 899.

feebly continued for some time after apparent death, in 17;—liquid, in 14; —in the state of soft coagulum, or merely grumous and without fibrinous concretions, in 12;—partly liquid and partly coagulated, in 9;—and wholly or nearly deficient in the heart, in 6. In one instance only there were fibrinous concretions without cruor, that is, without liquid blood or bloody serum. Dr. D. next gives a tabular account of 35 cases of post-mortem examinations, made in the General Hospital at Fort Pitt, Chatham, from January to Sept. 1838. The various conditions in which the blood was found are still more minutely described in these cases, than in the preceding ones; but in none of either set does clear serum appear to have been discovered, except in one solitary instance, and under very peculiar circumstances; namely, in No. 16, a case of phthisis, wherein, says the author,—' a mass of fibrin in the right ventricle contained a collection of transparent serum. The mass was firmest externally. There was some crassamentum, fibrinous concretions, and a good deal of cruor in the right cavities of the heart. Two hours after examination, the cruor was found jellied. After twenty four hours the coagulum had contracted, and serum had separated. When broken up and agitated, some air was given off.' "*

The testimony of Mr. Paget is still more precise:

"In all cases it must be remembered that the coagulation which takes place in the body is much slower than that which ensues in blood drawn from it, either during life or after death; so that a quantity of uncoloured fibrine is found in the heart and uppermost vessels of the dead body in many cases, in which it is most probable that, had the blood been drawn during life, it would not have presented a buffy coat. In the majority of cases, the blood does not coagulate in the body for the first four hours after its rest has commenced. In many it remains fluid for six, eight, or more hours, and yet coagulates within a few minutes of its being let out of the vessels. But, as this greater slowness of coagulation is common to all, it is not material in a comparison of the blood of the dead with that of the living."† P. 404.

Mr. Paget, in a letter addressed to our author subsequently to the publication of these remarks, observes:—"I have never found clear serum, such as I could suppose to be separated from the blood in its coagulation, collecting in any part of the body after death," *i. e.* as long as the blood remained within its natural receptacles. The following passage from Dr. Carpenter's excellent work on Human Physiology contains an apposite illustration of this point.

"Instances occasionally present themselves in which the blood does not coagulate after death, and in most of these there has been some sudden and violent shock to the nervous system, which has destroyed the vitality of solids and fluids alike. This is generally the case in men and animals killed by lightning, or by strong electric shocks, and in those poisoned by prussic acid, or whose life has been destroyed by a blow on the epigastrium. It has also been observed in some instances of rupture of the heart, or of a large aneurism near it, and a very interesting phenomenon then not unfrequently presents itself; the coagulation of the blood which has been effused into the pericardium, (the effusion having taken

---

* Researches Physiological and Anatomical. Vol. ii., pp. 190-213.

† London Medical Gazette, for 1840. Vol. i, p. 618.

place during the last moments of life,) whilst that in the vessels has remained fluid."* P. 150.

The various statements now made amply warrant the conclusion that the separation of the blood into serum and crassamentum seldom if ever takes place completely, as long as it remains in its natural receptacles, these being at the time in a normal state.

Dr. Stroud suspects, and with reason, that, in several cases of Rupture of the Heart on record, this separation had really taken place, although it is not specified in the account given of the appearances found on dissection. After alluding to three cases, related by Dr. Abercrombie in the first volume of the *Transactions of the Medico-Chirurgical Society of Edinburgh*, he quotes one that is recorded by Dr. Thurnam in the *Medical Gazette* for 1838. This was "'a case of rupture of the heart from external violence, but without any penetrating wound. The pericardium contained several ounces of serum and coagulated blood. There was a considerable rupture of the right auricle, and a smaller one at the apex of the heart.' The same gentleman mentions an instance 'of spontaneous rupture of the right auricle and ventricle, attended with great and general softening. The pericardium was filled with liquid blood,'—coagulation having apparently been prevented by the feebleness of the heart's action, which is usually attended with a corresponding condition of the blood."

Dr. Elliotson† has related the case of a female, who died suddenly with severe pain in the region of the heart. The pericardium was found distended with clear serum, and a very large coagulum: there was a rupture of the aorta near its commencement. In the case related by Dr. Fischer, and which was occasioned, it is said, by the slow operation of continued grief, it is stated in the account of the dissection:—"On puncturing the pericardium, which had the appearance of being distended by a substance of a dark blue colour, a quantity of reddish fluid escaped, and afterwards florid blood to the amount of two or three pounds. The membrane was then slit up, and the heart seen surrounded by a coagulum more than three pounds in weight. This having been cleared away, a rupture was discovered in the aortic [left] ventricle, which extended upwards from the apex, about an inch and a half on the external surface. The internal wound was found but about half-an-inch in length, and its lips [were] at least as wide again asunder as those of the external breach."‡

Dr. Townsend|| of New York mentions the case of a young female, who died suddenly in consequence, it was believed, of anguish of mind. "On dissection, the sac of the pericardium was found filled with about ten

---

* The contrast between the state of the blood within the cavities of the heart, and of that which was extravasated (into the pleura?), is well marked in one of Morgagni's cases. The quantity of limpid serum was so great that, on first opening the chest, it was believed to be a case of dropsy. But, on further examination, a large quantity of coagulated blood was found *infra aquam*, and the real disease was discovered to be an aneurism of the aorta, which had given way.

† Lumleyan Lectures on Diseases of the Heart, p. 30.

‡ London Medical Repository, vol. xi. pp. 422-427; and vol. xii. pp. 164-168.

|| On the Influence of the Passions in the production and Modification of Disease, pp. 51-56.

ounces of coagulated blood, and two of serum. The heart, on all sides covered by it, was of the ordinary volume, but much loaded with fat. At the summit of the aortic [left] ventricle was discovered the breach, from which the effused blood had issued. It was irregularly lacerated, and measured about half-an-inch in diameter."

Dr. Williams, of Southampton, communicated to our author the particulars of a somewhat similar case :

"R. W., a labourer, aged fifty-six years, had generally enjoyed good health but for ten years had suffered great despondency of mind, owing to the unfaithfulness of his wife. About six months before his death he was troubled with severe cough, which came on in paroxysms, generally at night and early in the morning, and after a fit of this kind was found one morning dead. A post-mortem examination took place in the presence of Mr. Boulton, surgeon, of Leamington. On opening the chest, the bag of the pericardium appeared much distended with fluid, and was of a dark blue colour. On cutting into it, a pint at least of transparent serum issued out, leaving the crassamentum firmly attached to the anterior surface of the heart. On further examination to ascertain the source of this hæmorrhage, we found the left ventricle, from the origin of the aorta downwards to within an inch of the apex, ruptured. The heart appeared in no way disorganized, there was no softness of its walls, the internal membrane was healthy, and so were the valves of each cavity." P. 100.

Reference may also be made to a case recorded by Mr. Adams,* of a man who died with severe cardiac symptoms, after great agony of body and mind. "The pericardium was found distended, and emitted when divided a quantity of serous fluid ; but the heart was entirely concealed by an envelope of coagulated blood in three distinct layers, owing to rupture of the left ventricle close to the septum, and nearer the apex than the base of the heart."

In a case of rupture of the right auricle of the heart from violence, recorded by Ludwig in his *Adversaria,* it is stated :—"the pericardium was so distended by a large quantity of transparent serum and coagulated blood as to push the lungs upwards. The yellowish serum contained in its cavity exceeded half-a-pound." The Commentaries of the Academy of Bologna, for 1757, contain an account of a man who died suddenly. In addition to other lesions observed in the body, a small rupture was found in the left ventricle of the heart, and the pericardium was so distended as to occupy a third part of the cavity of the chest. On opening it, a large quantity of serum was discharged, and two pounds of clotted blood were seen adhering to the bottom.

Besides the cases now quoted, Dr. Stroud refers to, and briefly describes, several others ; viz. : one related by Mr. Watson in the London Medical Repository for 1814 ; two related in the London Medical and Physical Journal for May 1822 and April 1826 ; one in Wheeler's Manchester Chronicle for Nov. 22, 1834 ; one in the Medico-Chirurgical Review for 1836 (the case of the late Sir David Barry) ; one in the Edin. Medical and Surgical Journal for January 1843 ; one in the Dublin Medical Transactions for 1830 ; and two very interesting cases by Dr. France, in a recent number of the Guy's Hospital Reports. In all these instances, the extra-

* Journal of Morbid Anatomy, Ophthalmic Medicine, &c. Art. v.

vasated fluid is stated to have been distinctly separated into its crassamentum and serum, *blood* and *water*.

Before dismissing the pathological subjects that have been engaging our attention, we may state that it has been asserted that a quantity of blood, or bloody fluid, has been found within the pericardium, even when there was no discoverable rupture of the heart or large blood-vessels. Two such cases are related in the German Ephemerides.

"Both the subjects were robust soldiers who died of excessive joy, in whose bodies no morbid condition was afterwards found, except a large quantity of clotted blood in the pericardium, by which the action of the heart had been suppressed. The latter author ascribes the effect to sudden distension of the exhalants opening on the inner surface of the membrane. This would correspond to the manner in which bloody sweat is produced; but, as the exhalants of the pericardium are very inferior both in size and activity to those of the skin, it is more probable that in such cases the effusion is due either to rupture of some of the nutrient vessels of the heart itself, termed its coronary vessels, or to hemorrhage from without, penetrating by a minute or circuitous passage into its capsule. Such at least is the opinion of Morgagni, Zecchinelli, and other anatomists. Of blood thus finding its way into the pericardium by a small aperture, which, without great attention might easily escape notice, the former gives several examples; and in the Ephemerides, Dr. Daniel Fischer mentions the case of a soldier who died suddenly after eating a hearty dinner, and in whose body the only morbid appearance discovered on inspection was,—'the pericardium filled and distended with very fluid and florid blood. The membrane having been divided longitudinally, in order to trace more exactly the source of the hemorrhage, this was found at the base of the heart, where a branch of the coronary artery had ruptured, and from which blood was still actually flowing.'"* P. 92.

Before proceeding to shew the bearing of the facts and reasonings hitherto adduced upon the main subject of our author's argument, we shall here introduce, for a reason that will be readily understood, a few remarks upon that mode of capital punishment that was resorted to in the case of our Lord.

It would seem that Crucifixion was practised among many nations from the remotest antiquity. The earliest instance on record is probably that of the chief baker of Pharaoh: for, although we read in our Bibles that he was *hanged*, Josephus expressly says that he was *crucified;* and we know that these two words are often exchangeable in the Scriptures, the one for the other. Most persons imagine that crucifixion was a Jewish mode of putting to death. Not so. The Jews only crucified the dead bodies of those who had been stoned for blasphemy: hence it was that the "nailing to a tree" was deemed by them as so peculiarly "accursed." Moreover, the Mosaic law enjoined that the body, so exposed, should be taken down before the sunset of the day on which the criminal had been slain. It is always to be remembered that Christ was condemned by a Roman tribunal, and was put to death by Roman law, although the guilty instigators of the

---

* "Ephemerid. Acad. Natur. Curios. Dec. iii. Ann. 9 and 10,—p. 293;—Ibid. Edit. 2da. vol. v. pp. 141, 142;—Matt. Van. Geuns, De Morte Corporea, &c. p. 591;—Zecchinelli, Sulla Angina del Petto, &c. vol. i. pp. 95, 96;—Thurnam, in Lond. Med. Gazette, 1838, pp. 813—817;—Curling, ibid. pp. 894, 895;—Fitzpatrick, in Lond. Med. Repository, vol. xvii. pp. 295—298.

act were those of his own nation. The Jewish Rulers were the accusers, Pilate was the judge, and the soldiers were the executioners. Now crucifixion was in common use among both the Greeks and the Romans, more particularly in the case of their slaves when convicted of a capital offence. It was consequently regarded as the most ignominious and disgraceful of all punishments. Everything was thus combined to render the death of the Saviour accursed in the sight of the people. He suffered as the vilest of malefactors according to the law of the heathen masters of Judea, for alleged *sedition* against the authority of Cæsar; while, at the same time, his being " nailed to the tree" represented and fulfilled, in the eyes of the Jews, the divine malediction that was always associated in their minds with the peculiar punishment for *blasphemy*—the pretext, it will be remembered, on which they sought His life.

Crucifixion appears to have continued in force among the Romans until the time of Constantine, the first Christian Emperor, who abolished it throughout his dominions. " He would not suffer the instrument of our salvation," says Crevier,* " to be dishonoured by any use, not only profane, but capable of making men look upon it with horror. He thought it indecent and irreligious that the cross should be employed for the punishment of the vilest offenders, whilst he himself erected it as a trophy, and esteemed it the noblest ornament of his diadem and military standards."

That death by crucifixion was usually protracted and lingering is not only abundantly testified by history, but is strictly in accordance with what might be anticipated from the very nature of the punishment. It will serve to give the reader a more correct notion of it, if we insert the following particulars respecting the construction of the Cross, as used in ancient times.

" The cross consisted of a strong upright post, sharpened at the lower end by which it was fixed in the ground, having a short bar or stake projecting from its middle, and a longer transverse beam firmly joined near its top. As the middle bar, although an important appendage, has been almost universally overlooked by modern authors, it will be proper here to insert the account given of it by some of the early fathers of the church, and founded on personal observation. —' The structure of the cross,' says Irenæus,—' has five ends or summits, two in length, two in breadth, and one in the middle, on which the crucified person rests.'—Justin Martyr, in like manner, speaks of—' that end projecting from the middle [of the upright post] like a horn, on which crucified persons are seated;' —and the language of Tertullian, who wrote a little later, exactly corresponds.— ' A part, and indeed a principal part of the cross is any post which is fixed in an upright position; but to us the entire cross is imputed, including its transverse beam, and the projecting bar which serves as a seat.'†—The criminal condemned to this dreadful mode of death, having first been scourged, was compelled to carry the cross on his shoulders to the place of execution, a circumstance which implies that the scourging was not excessively severe, and that the dimensions of the gibbet did not in general much exceed those of the human body. On arriving at the spot he was stripped of his clothes; and after receiving a cup

---

* History of the Roman Emperors. Vol. x. p. 132.

† " Irenæus, Opera, p. 166;—Justinus Martyr, Cum Tryphone Judæo Dialogus, pp. 271, 272;—Tertullianus, Ad Nationes, p. 49; Adversus Judæos, p. 195."

of wine, sometimes medicated with a view to impart firmness or alleviate pain, was speedily nailed to the cross, either before or after its erection. In either case he was made to sit astride on the middle bar; and his limbs having been extended and bound with cords, were finally secured by large iron spikes driven through their extremities, the hands to the transverse beam, and the feet to the upright post." P. 36.

With respect to the degree and usual duration of the sufferings inflicted by this horrible punishment, Dr. Stroud remarks :—

"The bodily sufferings attending this punishment were doubtless great, but, either through ignorance or design, have been much exaggerated. The insertion of the cross into its hole or socket, when the criminal was previously attached to it, did not necessarily produce the violent concussion which has been supposed; and as the body rested on a bar, it did not bear with its own weight on the perforated extremities. At all events, there have been many examples of persons enduring these sufferings with the utmost fortitude, and almost without a complaint, until relieved from them by death. A fact of importance to be known, but which has not been sufficiently regarded, is that crucifixion was a very lingering punishment, and proved fatal not so much by loss of blood, since the wounds in the hands and feet did not lacerate any large vessel, and were nearly closed by the nails which produced them, as by the slow process of nervous irritation and exhaustion. This would of course be liable to variety, depending on differences of age, sex, constitution, and other circumstances; but for persons to live two or more days on the cross was a common occurrence, and there are even instances of some who, having been taken down in time and carefully treated, recovered and survived. In many cases death was partly induced by hunger and thirst, the vicissitudes of heat and cold, or the attacks of ravenous birds and beasts; and in others was designedly accelerated by burning, stoning, suffocation, breaking the bones, or piercing the vital organs."* P. 38.

Numerous instances might be adduced from the writings of Martyrologists in proof of the slowness of death from crucifixion—according to Origen and other early fathers, the sufferer usually survived about two days; —but we prefer to quote the following accounts from two works of recent date.

"'The capital punishments inflicted in Soudan'—observes Captain Clapperton writing in 1824,—'are beheading, impaling, and crucifixion; the first being reserved for Mahometans, and the other two practised on Pagans. I was told, as a matter of curiosity, that wretches on the cross generally linger three days before death puts an end to their sufferings.'—When describing the punishments used in Madagascar, the Rev. Mr. Ellis remarks,—'In a few cases of great enormity a sort of crucifixion has been resorted to; and in addition to this, burning or roasting at a slow fire, kept at some distance from the sufferer, has completed the horrors of this miserable death...... In the year 1825 a man was condemned to crucifixion who had murdered a female for the sake of stealing her child. He carried the child for sale to the public market, where the infant was recognised, and the murderer detected. He bore his punishment in the most hardened manner, avenging himself by all the violence he was capable of exercising upon those who dragged him to the place of execution. Not a single groan escaped him during the period he was nailed to the wood, nor whilst the cross

---

* "Claudius Salmasius, De Cruce, &c., pp. 229—340, &c.;—Justus Lipsius, De Cruce, pp. 98—109, &c.; Dr. Adam Clarke, The New Testament, with a Commentary, &c.; Comment on Matt. chap. 27, v. 35."

was fixed upright in the earth. The wooden frame used in the place of a cross resembles a gallows. To this the malefactor is nailed whilst it remains flat upon the earth, after which it is lifted up with its miserable burden, and fixed in two holes made in the ground for the purpose. Here the sufferer is kept until he dies of cold, hunger, or agony. Some criminals after being nailed to the frame, have remained for hours for the gaze of the multitude. A fire has oftentimes been placed to windward of them, by which they and the cross have been consumed together.' " P. 42.

Mr. Slade also, in his record of Travels in Turkey, Greece, &c., gives the following horrible account of the execution at Constantinople of a captain of banditti, a few years ago:

" As a preparatory exercise, he was suspended by his arms for twelve hours. . . . . The following day a hook was thrust into his side, by which he was suspended to a tree, and there hung enduring the agony of thirst till the third evening, when death closed the scene; but before that about an hour the birds, already considering him their own, had alighted on his brow to peck his eyes. During this frightful period he uttered no unmanly complaints, only repeated several times,—' Had I known that I was to suffer this infernal death, I would never have done what I have. From the moment I led the klephte's life I had death before my eyes, and was prepared to meet it, but I expected to die as my predecessors, by decapitation." P. 43.

From what has now been stated, it may be very reasonably inferred that the death of Jesus, within six hours after He was nailed to the "accursed tree" was not, as is frequently believed, the result of extreme bodily torture, and therefore, as our author observes, that, " although the ordinary sufferings of crucifixion contributed to his death, they were not its immediate cause." It is certain that the byestanders, and others engaged in the dreadful act, were surprised at the suddenness of the Saviour's death; for we find that Pilate scarcely believed that such could be the case, when Joseph applied to him to take the body down from the cross.*

Admitting, then, that there was something unusual and extraordinary in the quickness with which the sufferings of our Lord came to a close, we now proceed to enquire, with all due reverence, whether any probable explanation can be offered to account for it. And here we must first remark that there is nothing in any of the sacred narratives, to make us believe that the human nature of the sufferer was brought to the verge of exhaustion by the bodily and mental sufferings—however great these may have been—which He had undergone, during the eighteen hours that elapsed from his agony in the garden up to the moment that preceded his dissolution. Commentators have repeatedly dwelt upon the circumstances of his crying *with a loud voice* " My God, my God, why hast Thou forsaken me!" of his then saying " I thirst," and receiving the vinegar that was offered to Him; and, last of all, of his again crying in a loud tone, " It is finished; Father, into thy hands I commend my spirit,"—as being utterly irreconcileable with the supposition that life was at its last ebb, from the extinction of vital energy. These were evidences and signs—to use the language of an intelligent and pious writer—that " his life was whole in Him and nature strong. The voice of dying men is one of the first things that fails.

---

* St. Mark, ch. xv., v. 44.

With a panting breath and faltering tongue, a few broken words are hardly spoken, and more hardly heard; but Christ, just before He expired, spoke like one in his full strength, to shew that his life was not forced from Him, but was freely delivered by Him into his Father's hands, as his own act and deed."*

One of two explanations must therefore be adopted to account for the suddenness of his dissolution; either that the Saviour, seeing that all things were now fulfilled, spontaneously, and by an act of His own divine will, yielded up His life; or that (not, we need scarcely say, independently of, but only in accordance with, His permission) some mortal lesion of a vital organ of his human frame suddenly supervened, and was the immediate and, so to speak, the physical cause of his death. Unquestionably the first of these opinions has been that which has generally been received by biblical commentators, and also, we should think, by most simple readers of the sacred narrative. It has this advantage, that it seems, at least upon first thought, to be most consonant with various expressions used by Christ himself in reference to his death: such as in that remarkable passage in the Gospel of St. John, where He says, "I lay down my life that I may take it again. No man taketh it from me, but I lay it down of myself." Moreover, it has been argued that the words αφηκε (and παραδωκε) το πνευμα, used by St. Mathew and St. John, rendered in our version "yielded, and gave up, the ghost," clearly imply a voluntary dismission of His spirit; but then, be it remembered, that the other two evangelists merely say that He expired, εξεπνευσεν.

Dr. Stroud—while he recognises, as every sincere Christian must do—in all its plenitude and force, the sovereign power of the Divine sufferer to yield or to retain his life, and only after the most studious and devotional investigation of all the scriptural statements, whether in the way of narrative, of prophecy, or of doctrine, in reference to the death of Christ—adopts the second of the opinions alluded to above. The positive declarations that He was slain by his enemies, that He died the death of the cross, that He "became obedient unto death," that the Jewish rulers were "his betrayers and murderers," that they "slew the Prince of Life whom God raised from the dead," &c., intimate, he thinks, that the death was the result and consequence of the crucifixion, and not of any supernatural agency. Some learned divines have come to the same conclusion. Thus Bishop Pearson, in his Exposition of the Creed, observes:—"Should we imagine Christ to anticipate the time of death, and to subtract his soul from future torments necessary to cause an expiration, we might rationally say the Jews and Gentiles were guilty of his death, but we could not properly say they slew Him. Guilty they must be, because they inflicted those torments which in time death must necessarily follow; but slay Him actually they did not, if his death proceeded from any other cause, and not from the wounds which they inflicted."

And our author has the following very appropriate observations on the same subject.

"That it was in the power of Christ to avoid such a death, had He chosen to

---

* M. Henry's Exposition on the New Testament.

renounce the object of his mission, is evident amongst other reasons from his miraculous overthrow of the hostile band in the garden of Gethsemane; from his question to Peter,—'Thinkest thou that I cannot even now request my Father, and he would send to my aid more than twelve legions of angels? [but] how then would the Scriptures be fulfilled, [which declare] that thus it must be?'—and from his remark to Pilate,—'Thou wouldst not have had any authority at all against me, had it not been given thee from above.'—In all the scriptural allusions to this subject, the death intimated, although voluntary, is moreover represented not as self-inflicted, but as penal and vicarious. In the very passage which has been thus misinterpreted, the death encountered by the good shepherd for the safety of his flock is ascribed to the wolf from whom the hireling flees." P. 59.

As a matter of course, it would be wholly out of place here to enter more at length upon the very interesting, but mysterious, question alluded to. It is not susceptible of perfect solution, and must therefore be left to each individual enquirer for calm reflection in his own mind.

We are now prepared to follow our author in his application of the facts and reasonings adduced, in the early part of the article, to the illustration (if so it may be called) of the sufferings of our blessed Lord. And first of his *bloody sweat.* It will be remembered that, on the night before his Crucifixion, after celebrating the Passover with his disciples, and instituting that rite which was thenceforth to be commemorative in all ages of his death, and having commended himself, his followers, and his cause, in solemn prayer, to the Father, He went forth with his disciples to the Mount of Olives, and thence, across the brook Cedron, to the garden of Gethsemane. We then read that, when He arrived at this place, He began to be seized with consternation and anguish (ηρξατο εκθαμβεισθαι και αδημονειν), so that his soul was "exceeding sorrowful, even unto death;"—that He then withdrew about a stone's-cast from the three favoured disciples whom He had taken with Him, and fell upon his face, and prayed most fervently that, if it were possible, that hour might pass from Him—that, returning to them, He found them asleep, and, after exhorting them to watchfulness, withdrew, and prayed a second time that, if it might be, the cup of his bitter affliction might be removed from Him—that, again returning to them, and again finding them asleep, He went away and prayed a third time, using the same words, "Father, if this cup may not pass from me, except I drink it, Thy will be done;—and that then, an angel having appeared from heaven to strengthen him, He fell into an agony,* and prayed

---

* The import of the word 'agony' will be better understood from the following remark of Castello, in his Lexicon Medicum, 1746, on its Greek etymon. "Agonia, ἀγωνια, *angorem,* significat, et verbum ἀγωνιᾶν, juxta Galen, lib. 2, *de symptom. caus.* cap. 5, *ad finem,* affectum animi compositum ex ira et timore; illa quidem sanguinem et spiritum foras agente et fundente, hoc vero utrumque ad vitæ principium et interiora, cum refrigeratione eorum quæ in summo corpore sunt, reducente et contrahente. Derivatur ab ἀγὼν, *certamen,* lucta. Unde et ἀγωνια certamen quandoque significat........ Ad summam, *Agonia* significat in genere colluctationem diversorum affectuum animi inter se contrariorum."

In the case of our Lord, it most probably implies a violent struggle of conflicting emotions—of overwhelming grief at the sense of divine abandonment on the one hand, and of intense desire of working out, even at the cost of his own life, the salvation of the human race, on the other. It is emphatically called by the prophet, the "travail of his soul."

with greater intensity, and "his sweat was as it were great drops of blood falling down to the ground," or, as Dr. Stroud renders the words, "his sweat became as it were clots of blood dropping to the ground, (εγενετο δε ὁ ιδρως αυτου ωσει θρομβοι αιματος καταβαινοντες επι την γην)*—Truly, never was sorrow like unto His sorrow!

Such is the simple and impressive account of that awful hour, given in the writings of the Evangelists. The following passage will now enable the reader to judge of the manner in which our author comments upon it.

"The intense grief and consternation which the Saviour experienced at the commencement of his sufferings in the garden, and under the shock of which he fell prostrate to the earth, might possibly have destroyed him by simple exhaustion, but would never have produced the bloody sweat reported by St. Luke; who, independently of his guidance by the Holy Spirit, was, as a physician, peculiarly well qualified to notice and record such an occurrence. He therefore ascribes this sweat to a cause by which it is fully and solely explained, namely, the communication of supernatural strength;—'There appeared to him an angel from heaven, strengthening him.'—It was then that,—'falling into an agony, [Christ] prayed most earnestly, and his sweat became as it were clots of blood dropping to the ground;'—implying that he was no longer prostrate as at first, but on his knees. Attempts have been made to explain away the strong terms used by the evangelist, but they certainly denote a sweat mixed with blood in a half-coagulated state, so profuse as to fall from the head and neck, (the parts chiefly liable to be uncovered, and from which sweat of any kind is most readily furnished,) in thick and heavy drops to the ground. Unless St. Luke meant to convey this meaning, his employment of such expressions is unaccountable. The fact is well stated by M'Lean.—[Christ] 'is said to be *in an agony*. An agony is a conflict of nature in the extremity of distress. The Lord was now bruising him, and putting him to grief. So great was the agony and conflict of his soul, that it produced the most wonderful effect upon his body; for we are told that—'his sweat was as it were great drops of blood falling down to the ground.'—A common sweat in the open air, and exposed to the cold damp of night, when those within doors required a fire of coals to warm them, must have been the effect of very great fear and agony. What then must his agony have been, which induced a bloody sweat, and so copious as to fall down in great drops to the ground?'—It was then that, as intimated by the Apostle Paul,—'he offered prayers and supplications, [accompanied] with tears and loud cries, to him who was able to save him from death, and was heard on account of his pious fear;'—in other words, these peculiar and overwhelming sufferings were by divine interposition suddenly terminated, leaving him with restored strength, ready to undergo the trials which next awaited him." P. 116.

It is unnecessary to do more than merely allude to the perfect calmness and meek composure with which He endured the indignities and sufferings of the mock trial, the buffetings and scourgings of the Roman soldiers, the blasphemous reproaches and revilings of his own countrymen, the labour of carrying the cross, the preparations for execution, and, lastly,

---

* The Greek word θρομβος is usually interpreted by the Latin one, *grumus.* Schleusner, in reference to it, says:—"In scriptis medicorum Græcorum admodum frequenter vox θρόμβος de gutta spissi et coagulati sanguinis, et de sanguine coagulato in universum usurpatur. v. c. Dioscorid. I, c. 102; Hesych. θρόμβος: αιμα παχὺ, πεπηγὸς, ὡς βουνοι. Confer. Faesii Œcon. Hippocr. p. 167." Dr. Stroud remarks, that "the force of the term ώσει, frequently used by St. Luke in a similar sense, evidently is that Christ's sweat on this occasion consisted of clotted blood, not pure, but mixed with the usual watery liquid."

the pains and torture of crucifixion itself. Not a word of complaint or impatience escaped his lips; "He was brought as a lamb to the slaughter, and as a sheep before her shearers is dumb, so He opened not his mouth." For the first three hours that He hung upon the cross all was silent and still on his part, save when He prayed for forgiveness to his murderers, when He gave the penitent thief a divine assurance of pardon and acceptance, and when He committed his widowed mother to the care of the beloved disciple. It was not until the sixth hour (noon) when the sun became obscured, and blackness overspread the earth, in attestation, as it were, of the hiding of his Father's countenance, and of the consequent renewal of that dark and overwhelming agony of mind which He had experienced on the preceding night, and which would then apparently have proved fatal, had not supernatural aid been granted, and the duration of the fearful conflict been limited to one hour.

"On both occasions," says our author, "these sufferings were distinguished from all others, by beginning and ending abruptly, as well as by their peculiar circumstances and effects. On both occasions, the gloom which oppressed the Redeemer's soul was by divine appointment accompanied with external darkness, as its appropriate sign and illustration. When He was in the garden the preceding evening, it appears from astronomical calculation that the paschal full moon underwent a natural eclipse; on which account, perhaps, the numerous party which went forth to seize him were provided with lanterns and torches. Twelve hours later on the same day, according to the Jewish mode of reckoning, a preternatural darkness overspread the whole land, from the sixth to the ninth hour." P. 120.

During this fearful interval, no intercourse took place between the suffering victim on the cross and the byestanders around; and a solemn pause in the evangelical narrative concurs with other circumstances to intimate that He was again enduring the peculiar sufferings of Gethsemane. It was then that, to use the language of inspiration, He "trod the wine press" of his Father's wrath, "alone," unstrengthened by heavenly aid, rejected and despised by the very world that He came to save, and exposed, at the same time, to the sharpest assaults of all the powers of darkness. At length, his human nature gave way under the anguish of the conflict, and the cord of life was snapped at the moment of his intensest suffering. Every thing thus seems to indicate that there was a sudden rent of the agonised tabernacle of life; and what more likely, we would reverentially ask, than that "the pitcher was broken at the fountain, and that the wheel was broken at the cistern"? But, without indulging this thought any further, let us again follow the sacred narrative. It was about three o'clock in the afternoon, the hour of sacrifice and just three hours before the commencement of the Jewish Sabbath, that the Saviour expired. Now, as we have already observed, the Mosaic law required that, before the Sabbath began, the crucified persons should be dispatched and removed. The Jews therefore applied to Pilate for permission to do this, and it was accordingly granted. It was then probably between four and five o'clock when the Roman soldiers came, and brake the legs of the two malefactors who had suffered along with Jesus.

"On finding Him already dead, they abstained from offering this needless violence to his corpse; but, as if to make sure, one of them with a spear pierced pierced his side, whence, says the beloved disciple, an eye-witness of the transaction,—'immediately there came forth blood and water,'—and with peculiar solemnity remarks that the whole took place under the superintendence of divine providence, in fulfilment of two ancient prophecies concerning Christ, one of which declared that none of his bones should be broken, and the other, that the guilty people of Israel should look on him whom they had pierced." P. 123.

To account for the flow of "the blood and water" from the spear-wound, many conjectures have at different times been proposed. The ancient commentators generally "had recourse to their favourite expedient of miraculous interposition, designed, as they imagined, to convey important symbolical instruction." By several of the modern ones, it has been "ascribed to serous effusion either into the pericardial or pleural sacs, naturally produced by that extreme debility which they suppose to have attended the Saviour's death." With respect to the first of these explanations, it would be altogether inexpedient here to examine or discuss it. As to the second, we shall now briefly state the reasons which Dr. Stroud considers to be conclusive against its adoption.

It will be remembered that the wound was on the left side, just in the region of the heart; and indeed it was with the view of insuring the death of the sufferer that the Roman soldier thrust the spear into his side. He knew nothing of the ancient prophecy that the Jews should "look upon Him whom they had pierced;" it was merely in the fulfilment of his stern duty to see that the criminals were put to death; else his own life might be made a forfeit for his negligence.

"The Roman practice of despatching in some instances crucified persons by breaking their legs, stabbing them with swords or spears, &c., is well known, and, as above noticed, has been fully described by Salmasius, Lipsius, Bosius, and others. When the soldier, therefore, pierced the side of Christ, he did nothing more than what was usual, and, having such an object in view, would naturally inflict a decisive wound, that is, a stab to the heart. This opinion has accordingly been adopted by a great number of theological writers, many of whom are cited by Thomas Bartholinus, a Danish physician, who, however, in an express treatise on the subject follows the guidance of his father Caspar, and objects to this opinion for no better reason than that, when speaking of the wound, and of the scar which remained after Christ's resurrection, the evangelist John mentions the side only, and not the heart. As a faithful witness of the transaction, John of course relates only what he saw, but leaves his readers to draw a rational inference from the facts described, which can be none other than that here stated."* P. 131.

But as it has very generally been admitted that the wound penetrated to the heart, and was designed to prove fatal had life still continued, we shall pass on at once to consider what is the most probable cause of the flow of "blood and water," which followed the thrust of the spear. The opinion of Bartholinus that, the latter issued from the bag of the pleura, has been partially at least adopted by several writers. Thus the Rev. Mr. Hewlett, in his notes upon the Gospels, expresses himself to this effect:

---

* "Thomas Bartholinus, De latere Christi aperto, &c., pp. 17—22, 45, &c.; Idem. Epistola ad Hieron. Bardium, pp. 565—570.—Acts, chap. 12, v. 18, 19."

"'Medical writers afford numerous instances of a large effusion of bloody lymph into the cavities of the pleura, from diseases of the lungs, and in cases of violent death with long struggling.......... A skilful and learned physician informed the editor that in cases of violent and painful death there is usually an effusion of lymph, or of lymph mixed with blood, into the cavities of the chest and abdomen......... It is, however, reasonable to acquiesce with those who are of opinion that the evangelist here intended to express more than a pathological fact.'—The physician meant in this passage was no doubt the late Dr. Willan, who—in his 'History of the Ministry of Jesus Christ,'—makes a similar remark, equally indicative of doubt and uncertainty.—'We have instances of watery effusion into the cavities of the pleura to a considerable amount, in cases of violent death with long struggling......... The phenomenon here mentioned by the evangelist is generally looked upon as miraculous.' "* P. 135.

Some have supposed that the blood came from the heart and the water from the pericardium, both being pierced by the thrust of the spear. The elder Gruner thus comments upon the subject:

"It was doubtless the left side that was pierced by the soldier's spear. According to the testimony of John, immediately after the infliction of this wound there flowed out blood and water. Such an effusion could scarcely have taken place, except on the left side, under which, besides the lung, lies the pericardium full of water when a person dies after extreme anxiety, as likewise the heart, connected with the arch of the aorta. The lung slightly wounded might have yielded a little blood, but certainly not water. That conjecture is, therefore, the most probable, and the most in accordance with forensic medicine, which derives the blood from the [left] ventricle of the heart, and the water from the pericardium." P. 136.

In a special treatise on the cross and crucifixion, Kipping draws the same conclusion, with the exception of regarding the water poured out on this occasion as naturally contained in the pericardium. Bishop Watson too, in his Apology for the Bible, has adopted the same view. But then, as Dr. Stroud very justly remarks, the quantity of serum usually found in the pericardium after death is so small that, "in a case like that under consideration, it would have been absolutely imperceptible." Haller states that a small quantity, not exceeding a few drachms, has frequently been found in the pericardium of executed persons; and John and Charles Bell deny the occurrence altogether, except under very peculiar circumstances: "If," these eminent anatomists observe, "a person have laboured under a continued weakness, or have been long diseased, if a person have lain long on his death-bed, if the body have been long kept after death, there is both a condensation of the natural halitus in all the parts of the body, and an exudation of thin lymph from every vessel, there is water found in every cavity from the ventricles of the brain to the cavity of the ankle-joint, and so in the pericardium amongst the rest. But, if you open a living animal, as a dog, or if you open suddenly the body of suicides, or if you have brought to the dissecting-room the body of a criminal who has just been hanged, there is not in the pericardium one single particle of water to be found."

In the natural state, the quantity of the pericardial fluid is certainly very

* Hewlet's Bible, &c.; Notes on John, chap. 19, v. 34, and Acts, chap. 1, v. 18;—Dr. Willan, History of the Ministry of Jesus Christ, &c. p. 195.

small; but after some forms of violent death, more especially when this has been attended with obstructed respiration, there is often rather more; and then it is either pure or tinged with blood. "An effusion of the latter kind is said to have been noticed in stags killed after a hard chase; and in some rare instances of sudden death, occasioned by strong mental emotion, the pericardium has been found distended with blood, owing probably, as Morgagni suspected, to organic disease, and the rupture of vessels; but for the statement of the Grüners that, after death accompanied with anxiety, the pericardium is full of water, there is no evidence."*

Besides, if such an effusion take place slowly and gradually, the fatal result will be not sudden or unexpected, but lingering, and preceded by symptoms of oppression and suffocation. But this pathological objection has little weight, compared with that derived from the simple narrative of the sacred text; for is it not obvious that the discharge of blood and water must have been considerable, and the distinction between the two well marked, to have been clearly observed and so emphatically mentioned by St. John? Dr. Stroud remarks upon this point:—

"Bloody serum, whether originally effused in that state, or resulting from subsequent mixture, would not have presented this character; for it would neither have issued rapidly, nor in sufficient quantity, nor would its distinction from ordinary blood have been so striking as to have attracted the attention of an uninformed, and somewhat distant spectator. Moreover, unless blood has been previously extravasated, little or none can by any kind of wound be extracted from a dead body, except by the action of gravity, the heart being usually empty, or, if otherwise, devoid of power to expel its contents. This important fact, overlooked by most other writers, was perceived and acknowledged by the Grüners, who nevertheless failed to discover the true explanation, and were induced to adopt the inadmissible opinion, that Christ was not actually dead when pierced by the soldier's spear, but merely in a faint and languid condition, which allowed the heart to act feebly, and, on being wounded, to pour forth its blood, preceded by the water, which they suppose had previously collected in the pericardium." P. 141.

The conclusion which our author deduces from the whole inquiry, of which we have given an abstract in the preceding pages, is given in the following words:

"It may, therefore with certainty be affirmed that, between the agony of mind which the Saviour endured in the garden of Gethsemane and the profuse sweat mixed with clotted blood which so rapidly followed it, violent palpitation of the heart must necessarily have intervened; this being the only known condition which could have been at once the effect of the former occurrence, and the cause of the latter. In like manner, when on the cross this agony was renewed, and by the addition of bodily suffering was increased to the utmost intensity, no other known condition could have formed the connecting link between that mental anguish and His sudden death,—preceded by loud exclamations, and followed by an effusion of blood and water from His side when afterwards pierced with a spear—than the aggravation even to rupture of the same violent action of the heart, of which the previous palpitation and bloody sweat were but a lower degree and a natural prelude. If, whilst every other explanation hitherto offered has been shewn to be untenable, the cause now assigned for the death of Christ,—namely, Rupture of the Heart from Agony of Mind—has been proved to be the result of an actual power in nature, fully adequate to the effect, really present without counteraction, minutely agreeing with all the facts of the case, and necessarily implied by them, this cause must according to the principles of inductive reasoning be regarded as demonstrated."

It is not without interest to learn that some of the commentators, ancient as well as modern, on the Gospels, had adopted views which, with the exception of certain extravagant expressions and errors of detail in their exposition, will be found to be in strict accordance with our author's ideas. Want of space, however, utterly precludes our giving any quotations.

But what is of much greater importance than any merely human corroborative testimony is the circumstance of there appearing to be a distinct allusion, in several prophecies in the Scriptures of the Old Testament, to the more remarkable of the physical effects of the Saviour's sufferings, including the bloody sweat, the intense thirst, and finally the mortal rupture of the heart. In the 22nd Psalm it is said,—" I am poured out like water; my heart is like wax; it is melted in the midst of my bowels: my tongue cleaveth to my jaws;" and, in the 69th, we read that "reproach hath broken my heart." There is a still more striking intimation of the cause of death in that memorable prediction in the 53rd ch. of Isaiah, where we are told that " He poured out His soul (or, as Michaelis and some other distinguished Hebraists render it, *His life's blood*) unto death." When too we remember that, in all the types in the patriarchal and Mosaic dispensations pre-figurative of the death of the Messiah, the shedding of blood was a necessary accompaniment of expiatory sufferings, and when we think of the symbols of the bread broken and the wine poured out in the rite of the last supper, not to mention the rending of the veil of the temple, the cleaving of the rocks, and the opening of the graves at the very moment of the mortal struggle, does not Dr. Stroud's explanation gain more and more upon our convictions? and do we not feel that he is justified in saying that, " the peculiar cause of the death of Christ, which by a regular induction from the evangelical narrative has been ascertained as a fact, remarkably illustrates the entire series of types and prophecies relating to that solemn event, which could not indeed in any other manner have been completely fulfilled? These in turn, by their minute and perfect correspondence with the circumstances, afford, if that were necessary, an additional confirmation of the fact itself; and the whole transaction demonstrates with irresistable evidence the special interposition and superintendence of the Deity."

And now we must take leave of our author, but not without again expressing our high admiration of his character and attainments, and saying at the same time that, in drawing to a close the present series of this Journal—and with it the Medico-Chirurgical Review itself as a separate and independent publication—it gives us very sincere gratification to have it in our power to recommend such a volume as the present to the thoughtful perusal of all our readers.

